# Hospital Progress and Finance

**Published:** 1924-03

**Authors:** 


### HOSPITAL PROGRESS AND FINANCE.

### THE LISTER WARD AND LISTER'S CENTENARY.

Although the managers of the Glasgow Royal Infirmary, as we noted in our November issue, have decided to destroy the famous Lister Ward, and it is understood that tenders have actually been accepted for its demolition, Mr. James A. Morris, F.R.I.B.A., has followed his admirable "Humble Plea" for its retention with another pamphlet, believing that at the eleventh hour this disastrous decision might be averted. His new pamphlet, "A Further Plea," published by Messrs. Maclehose, Jackson and Co., at sixpence, though omitting the plans that showed at a glance the destruction of this public monument to be wanton and unnecessary, is yet telling enough. The plans for the new buildings will encroach far more on the courtyard than the Lister Ward, and, since this occupies the ground floor only, whereas rising staircases, says Mr. Morris, are shown upon the plans, the substituted buildings will have many more of the very disadvantages that were made the excuse for the destruction of the Lister Ward. It is difficult, then, to avoid the conclusion that the demolition of the Ward is the real but unavowed object in view, and since its preservation has been prayed for in all parts of the world, public opinion should insist upon delay and reconsideration.

### THE OPPORTUNITY THAT REMAINS.

Mr. Morris remarks that the managers have never asked the public for money to purchase another site, though the site in Parliamentary Road purchased for building a dispensary has not yet been used, and the offer of £2,000 for the reconstruction of the Lister Ward still holds good. Finally, in 1927, the centenary of Lord Lister's death will be celebrated, and the celebrations in Glasgow, at least, will seem little more than hypocritical if they follow so soon upon an act of vandalism. One chance remains. If the various memorialists would combine to make an address to the managers of the Royal Infirmary, asking that the Lister Ward should be left untouched until the centenary celebrations are over, it could hardly be refused, for pilgrims from all over the world will desire to have a final opportunity to examine the historic scene of Lister's labours. Mr. James Morris and other public-spirited men should seize this opportunity, the last that we may have, of securing on public grounds a stay of execution.

### A BIG SCHEME IN NORTH STAFFORDSHIRE.

Though, in consequence of the £32,000 raised last year by the special appeal, the North Staffordshire Infirmary is out of debt for the first time in its history, the ordinary income was still below the expenditure, and important extensions are required. A sum of £150,000 is immediately needed for a new laundry and boiler house, for 60 new nurses' and maids' bedrooms, lecture rooms, and dining-room and kitchen extensions. These are preliminary to a new ward unit with 100 beds and the necessary annexes, while the medical staff desires a new X-ray department. Toward all this £34,000 is in hand, and a convalescent home would relieve the pressure on the beds and avoid the present necessity of turning out patients at the earliest and least favourable moment. Besides this the out-patient department is clogged, and, in short, the Infirmary cannot cope with the present demands upon it. The new president, Mr. Samuel Clowes, has a big task in front of him, but it is satisfactory that the workers' contributions rose to over £20,000 last year, an increase of £4,600, and that the National Council of the Pottery Industry has formed a committee to establish a uniform scheme of support from employers and employed, which should bear its fruit before the end of the year. With the income tending to increase, and the debt removed, the time is favourable for a big extension, but the lesson of last year's appeal suggests that the money required will only be raised if the industries of the entire district make a special levy on themselves for the purpose, towards which a good send-off by a handful of wealthy donors would be the best incentive.

### THE SUSSEX PROVIDENT SCHEME.

This scheme which, since its start, has had a good many difficulties to contend with, seems to have taken root and to be spreading, if slowly. Last year it paid over to the Royal Sussex County Hospital some £2,200, and the Children's Hospital received £389. As the scheme was early in the field it is unfortunate that it has not yet realised the hopes of its founders, but all experience shows that once a start has been made success is usually steady and progressive. The County Hospital, largely through its active president, Mr. Harry Preston, has made heroic efforts to place its finances in order, and the restoration of confidence that these efforts have earned will react favourably on the Provident Scheme to which the hospital is so important.

### A REMEDY FOR RECOMMENDS.

The system of hospital notes, notoriously an abuse, which, though dying, still persists, has led in Birmingham to an attempt to modify the hardships that it entails. The Birmingham Citizens' Society began last year to advocate the pooling of those issued, and the Society has since received an increasing number from hospital subscribers on condition that it will investigate any cases that the subscribers send where recommendations are needed. The success of the plan depends upon its general adoption, and it is already found that subscribers will, as a rule, part with some of the notes to which their subscription entitles them, if not with all. The whole system is deplorable and out of date, and the attitude of mind that once delighted, or was supposed to delight, in petty patronage is now much less common. So long, however, as the note-system endures, sufferers seeking admission to hospitals will continue to be subjected to endless humiliations and delays in seeking for notes by visit or letter from hospital subscribers; and this can be much reduced if all the notes purchased, or a large proportion of them, are pooled by some central body. For this palliation the Birmingham Citizens' Society is to be thanked and deserves to be supported, though we have little doubt that Mr. Richard Clements, its general secretary, would agree that the pooling of hospital letters is only a temporary remedy for an obsolete abuse that should be abolished altogether.

---

### THE EARLIEST REPORT.

The report and accounts, published in book form, of the Manchester Royal Infirmary, were in the hands of the trustees seven days before the meeting was held, on February 8. In this respect the Infirmary believes itself to be unique, and the question arises—if a voluntary hospital of this size can furnish its subscribers with a report giving all the essential details of its work for the past twelve months by the end of January, why do not other similar institutions find it possible? With 614 beds at its disposal, the hospital has a daily average of 541 occupied. The number of in-patients dealt with is 11,198, and each patient remains in the hospital an average of seventeen days. A comparison of the seventeen items of income with those for 1922 shows an increase in all but one—the maintenance of war pensioners; and the expenditure shows a reduction under every heading except two. None the less, the excess of expenditure over income is £12,312. Despite recent efforts, the hospitals of Manchester are not nearly so well supported as they ought to be.

---

### THE AMERICAN HOSPITAL IN LONDON.

The Provisional Committee of the American Hospital in London has established itself at 56-60, Hallam-street, Portland Place, W. The contributions already received, we understand, justify an immediate beginning, and the number of beds will be increased as soon as may be. The patients will be confined to American citizens, and to the American wives of British subjects and their children. The committee expects the temporary plan to develop rapidly into an organisation large enough to meet all anticipated demands. The medical director is Mr. Philip Franklin. The American Consul-General (Mr. R. P. Skinner), the president of the American Chamber of Commerce (Mr. F. E. Powell), Mrs. Curtis Brown, President of the Amercican Women's Club, with Mr. Franklin, form the committee.

---

### EXTENSIONS AND A NEW HOSPITAL AT LEICESTER.

The Leicester Royal Infirmary has entered on a new phase of activity since the extensions, which have been in progress for three years, are practically complete. A significant sign is that with the 400 beds now available the waiting list is expected to be cleared by the end of June. The 166 bedrooms in the enlarged nurses' home are almost all occupied, and the out-patient department has been improved by a new entrance, new registration and waiting rooms, which Mr. Fielding Johnson, the chairman, has generously provided. Mr. Johnson has further announced that before long another hospital will be added to the city, and this is understood to be a further generous instance of his public spirit. A number of patients have been attending the out-patient department for insulin treatment, and Mr. Harry Johnson, the house governor, states that the National Health Insurance Committees are being looked to for recognition of the work done. Income and expenditure approximately balance at the round figure of £60,000, and the Hospital Saturday Fund is expected to raise a gross income of £36,000, a wonderful total. Moreover, support from the county areas is becoming well organised and general. The 150th anniversary fund raised £75,000 or three quarters of the total asked for, and it seems likely that the hospital will be able to support the heavy burden that these great additions entail.

---

### THE NEW BRADFORD INFIRMARY.

Sir James Hill, Bart., head of the Bradford firm of wool merchants, has given £30,000 toward the new Bradford Royal Infirmary, the building of which at Daisy Hill, on the city's outskirts, is expected to be begun during the present year. Sir James, who is trustee and honorary treasurer of the hospital, has taken a large part in the negotiations with the Bradford Corporation for the sale of the present hospital, which the hospital board has offered to sell for £100,000. Apart from these sums, £40,000 is already in hand toward the anticipated cost of £400,000 for the new Infirmary. The transfer of the institution has much in its favour. It would remove the hospital from a cramped site in the centre of the city with slums adjoining it on one side to open and rural surroundings, within reasonable access of the town; and Sir James Hill's generosity comes at a timely moment and will bring the scheme appreciably nearer fruition. Coming from a hospital worker, his example has additional value, and will encourage others to support the great transfer upon which so much thought has already been spent.

---

### SMALL MATERNITY HOSPITALS.

The need for a modern maternity hospital in Edinburgh has led to a discussion of the respective merits of large and small hospitals. The Edinburgh Royal Infirmary is contemplating a big scheme of expansion, in which a maternity block will play an important part. Meantime a small maternity hospital, the Elsie Inglis Memorial, Spring Gardens, is being built, and will probably be completed before the end of the current year. The advocates of the two schemes have been emphasising their respective advantages, but these do not seem to be mutually exclusive. There is, indeed, room for both. The proposed block will possess the advantages of centralisation for the convenience of patients and staff and the facility of teaching, but the needs of the entire city will hardly be met by this alone, and the supporters of the small hospital claim that its site was carefully chosen for the work it is designed to do.

